# Inflammatory marker profiles and in‐hospital neurological deterioration in patients with acute minor ischemic stroke

**DOI:** 10.1111/cns.14648

**Published:** 2024-03-03

**Authors:** Luo Yi, Zi‐Xiao Li, Ying‐Yu Jiang, Yong Jiang, Xia Meng, Hao Li, Xing‐Quan Zhao, Yi‐Long Wang, Li‐Ping Liu, Yong‐Jun Wang, Hong‐Qiu Gu

**Affiliations:** ^1^ Department of Neurology, Beijing Tiantan Hospital Capital Medical University Beijing China; ^2^ China National Clinical Research Center for Neurological Diseases, Beijing Tiantan Hospital Capital Medical University Beijing China

**Keywords:** acute ischemic stroke, cerebrovascular disease, inflammatory marker, neurological deterioration

## Abstract

**Aim:**

The aim of the study was to analyze the association between inflammatory marker profiles and in‐hospital neurological deterioration (ND) in acute ischemic stroke (AIS) patients.

**Methods:**

Data from patients with minor AIS from the Third China National Stroke Registry were analyzed. Inflammatory cytokine levels within 24 h of admission were measured. The primary outcome was in‐hospital ND (an increase in National Institutes of Health Stroke Scale score ≥4 from admission to discharge). Associations were evaluated using odds ratios (ORs) and 95% confidence intervals (CIs) derived from logistic regression models. Net reclassification improvement (NRI) and integrated discrimination improvement (IDI) were used to evaluate incremental predictive values.

**Results:**

A total of 4031 patients (1246 women, 30.9%) with a median age of 62 years were included. In‐hospital ND occurred in 121 patients (3%). Each standard‐deviation increase in interleukin (IL)‐6 (OR, 1.17 [95% CI, 1.06–1.31]) and high‐sensitivity C‐reactive protein (hsCRP) (OR, 1.43 [95% CI, 1.24–1.66]) levels was associated with increased in‐hospital ND risk. Incremental predictive values for adding IL‐6 (IDI, 0.012; NRI, 0.329) but not hsCRP levels to the conventional risk factors were found.

**Conclusion:**

In minor AIS, hsCRP and IL‐6 levels were associated with in‐hospital ND, including IL‐6 levels in prognostic models improved risk classification.

## Graphical abstract

Elevated hsCRP and IL‐6 levels indicated higher ND risk. Adding inflammatory cytokines significantly improved risk classification, with IL‐6 as the main contributor.

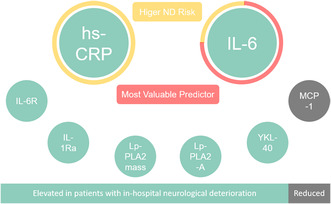

## 1 INTRODUCTION

The disease burden due to stroke is increasing worldwide and in China.^1^, ^2^ Neurological deterioration (ND), whether it occurs hours or weeks after stroke onset, is associated with adverse prognosis and death.^3^, ^4^, ^5^, ^6^ ND may not always reverse during hospitalization and can persist until discharge.^7^ Therefore, patients with a higher ND risk should be identified and prioritized to improve in‐hospital treatment strategy. Specifically, patients with minor stroke may receive less attention than those who exhibit more obvious symptoms of neurological impairment at admission.

Several inflammatory cytokines, including high‐sensitivity C‐reactive protein (hsCRP),^8^, ^9^ interleukin (IL)‐6,^10^ and lipoprotein‐associated phospholipase A2 (Lp‐PLA2),^11^ have been identified as independent predictive factors for functional worsening in previous studies. However, these studies often did not focus on cases of minor stroke,^8^, ^10^, ^12^, ^13^, ^14^, ^15^, ^16^, ^17^, ^18^, ^19^ failed to exclude the influence of thrombolysis therapy,^9^, ^12^, ^17^, ^20^ and were constrained by limited sample sizes and types of inflammatory biomarkers.^21^

Using data from the Third China National Stroke Registry (CNSR‐III), a large‐scale registry involving the centralized testing of multiple inflammatory biomarkers within 24 h of admission, we meticulously evaluated the associations of inflammatory biomarkers with in‐hospital ND in patients with minor stroke who did not undergo reperfusion therapy and assessed the incremental predictive values of these inflammatory biomarkers when added to traditional risk factors.

## 2 METHODS

The study was approved by the ethics committee of Beijing Tiantan Hospital (approval number: KY2015–001–01) and has therefore been performed in accordance with the ethical standards laid down in an appropriate version of the Declaration of Helsinki. Written informed consent was obtained from the patients or their legally authorized representatives.

### 2.1 Study design and participants

Data were obtained from CNSR‐III, a national prospective cohort of patients with acute CVDs for long‐term follow‐ups. Overall, 15,166 patients with ischemic stroke or transient ischemic attack (TIA) within 7 days of onset were enrolled in the registry. The CNSR‐III protocol has been previously described.^22^

Patients with minor AIS (National Institutes of Health Stroke Scale [NIHSS] ≤5, arrived within 24 h) were included in the current study. Patients who received thrombolysis or thrombectomy treatments, had missing NIHSS scores at admission or discharge, or missing data on inflammatory marker levels were excluded.

### 2.2 Data collection and measurement

Direct interviews and medical records included information on age, sex, current tobacco use, NIHSS score on admission, blood pressure, medical history, characteristics of infarction, and stroke etiology. Collected within 24 h of admission, blood samples were aliquoted, transported to the central laboratory of Beijing Tiantan Hospital in a cold chain, and stored. The levels of hsCRP, IL‐6, IL‐6 receptor (IL‐6R), IL‐1 receptor antagonist (IL‐1Ra), Lp‐PLA2 mass and activity (Lp‐PLA2‐A), YKL‐40/chitinase‐3‐like protein 1 (CHI3L1), and monocyte chemotactic protein 1 (MCP‐1) were tested centrally and blindly.

### 2.3 Outcome assessment

Outcomes were assessed by trained research coordinators through in‐person interviews at admission and discharge. The primary outcome was in‐hospital ND, defined as ΔNIHSS score ≥4 (ΔNIHSS = NIHSS score at discharge—NIHSS score at admission). This definition was used because we mainly focused on the development of potential improvement strategy of in‐hospital treatment for acute minor stroke.

### 2.4 Statistical analysis

Participants were dichotomized according to their in‐hospital ND status. Categorical variables are presented as frequencies and proportions in percentages. Continuous variables are presented as means with standard deviations and as medians with interquartile ranges for better explanation. To check the normality of variables, we used the Kolmogorov–Smirnov test and Q‐Q plot. For a large data set, *p* < 0.05 indicates statistical significance, but the result may not have clinical significance. Therefore, baseline characteristics and biomarkers were compared using the absolute standardized difference (ASD). An ASD ≥10% was considered clinically significant.^23^

The association between serum inflammatory parameters and ND status was assessed using unadjusted and adjusted odds ratios (ORs) and 95% confidence intervals (CIs) from multivariable binary logistic regression. Confounding variables adjusted for in the multivariate analyses were determined based on clinical experience and significance tests (Table 1). The first quartile was the reference group. Trend tests were performed using quartiles as ordinal variables. To assess the incremental predictive value of inflammatory markers in addition to conventional risk factors, C‐statistics, integrated discrimination improvement (IDI), and the net reclassification improvement (NRI) were applied. A two‐sided *p* < 0.05 was considered statistically significant. All analyses were performed using SAS software (version 9.4; SAS Institute).

**TABLE 1:** Demographic and clinical characteristics of patients by In‐hospital ND status.

Variables	No ND (*N* = 3910 [97.0%])	ND (*N* = 121 [3.0%])	ASD (%)
Demographics			
Age in years	62.1 ± 11.3	65.0 ± 12.0	24.9
Female	1199 (30.7)	47 (38.8)	17.1
Smoking	1276 (32.6)	27 (22.3)	23.2
NIHSS at admission	2.0 (1.0–4.0)	2.0 (1.0–3.0)	0.0
NIHSS			12.3
NIHSS ≤3	2899 (74.1)	96 (79.3)	
NIHSS 4–5	1011 (25.9)	25 (20.7)	
SBP	150.6 ± 22.1	154.7 ± 25.4	17.2
DBP	87.6 ± 13.2	88.6 ± 14.9	7.1
Medical history			
Prior stroke/TIA	978 (25.0)	27 (22.3)	6.4
Hypertension	2443 (62.5)	75 (62.0)	1.0
Diabetes mellitus	899 (23.0)	32 (26.4)	7.9
Lipid metabolism disorders	351 (9.0)	7 (5.8)	12.2
Prior CHD/MI	422 (10.8)	13 (10.7)	0.3
Atrial fibrillation/flutter	252 (6.4)	9 (7.4)	3.9
Heart failure	23 (0.6)		10.9
Peripheral arterial disease	42 (1.1)		14.7
Carotid stenosis	36 (0.9)		13.6
Infarction pattern			20.1
None	1076 (27.5)	28 (23.1)	10.1
Single infarction	1563 (40.0)	42 (34.7)	11.0
Multiple infarction	1227 (31.4)	49 (40.5)	19.0
Watershed infarction	44 (1.1)	2 (1.7)	5.1
Infarction circulation			10.1
None	1076 (27.5)	28 (23.1)	10.1
Anterior circulating infarction	1695 (43.4)	56 (46.3)	5.8
Posterior circulation infarction	959 (24.5)	31 (25.6)	2.5
Anterior and posterior circulatory infarction	180 (4.6)	6 (5.0)	1.9
Stroke etiology			27.0
LAA	808 (20.7)	37 (30.6)	22.8
CE	243 (6.2)	8 (6.6)	1.6
SVO	965 (24.7)	20 (16.5)	20.4
Other	1894 (48.4)	56 (46.3)	4.2
Onset to discharge days	13.0 ± 6.2	17.8 ± 13.3	47.0

Abbreviations: ASD, absolute standard difference; CE, cardioembolic stroke; CHD, coronary heart disease; DBP, diastolic blood pressure; LAA, large‐artery atherosclerosis; MI, myocardial infarction; ND, neurological deterioration; NIHSS, National Institutes of Health Stroke Scale; SBP, systolic blood pressure; SVO, small‐vessel occlusion; TIA, transient ischemic attack.

## 3 RESULTS

### 3.1 Baseline characteristics

Of 15,166 patients, 5886 with AIS over 24 h after onset, 2739 with moderate or severe stroke (NIHSS >5), 618 who received reperfusion treatments (including thrombolysis and thrombectomy), 8 with missing NIHSS records at admission or at discharge, and 1884 with missing inflammatory marker data were excluded. Thus, the final analysis included 4031 patients (Figure S1). And 43 patients lost during follow‐up with no specific reasons were recorded. Except for symptoms, lipid metabolism disorders, and atrial fibrillation/flutter, the baseline characteristics of the patients were similar with or without inflammatory markers. However, no apparent clinical significance was associated with the abovementioned differences (Table S1).

The median age of the patients was 62 years, and 1246 (30.9%) were women. Table 1 presents the baseline characteristics by ND status. The average NIHSS scores at admission and discharge were 2.17 ± 1.65 and 1.47 ± 2.10, respectively, with a mean ΔNIHSS of −0.70 ± 2.09. The average time between the disease attack and hospital discharge was 13.1 ± 6.6 days, and the total ND duration was 17.8 ± 13.3 days. Patients with ND tended to be older, tobacco users, had higher systolic blood pressure (SBP), and had a stroke etiology of large‐artery atherosclerosis (LAA) and small‐vessel occlusion (SVO) than patients without ND (Table 1).

### 3.2 Inflammatory profiles and in‐hospital ND

**FIGURE 1:**
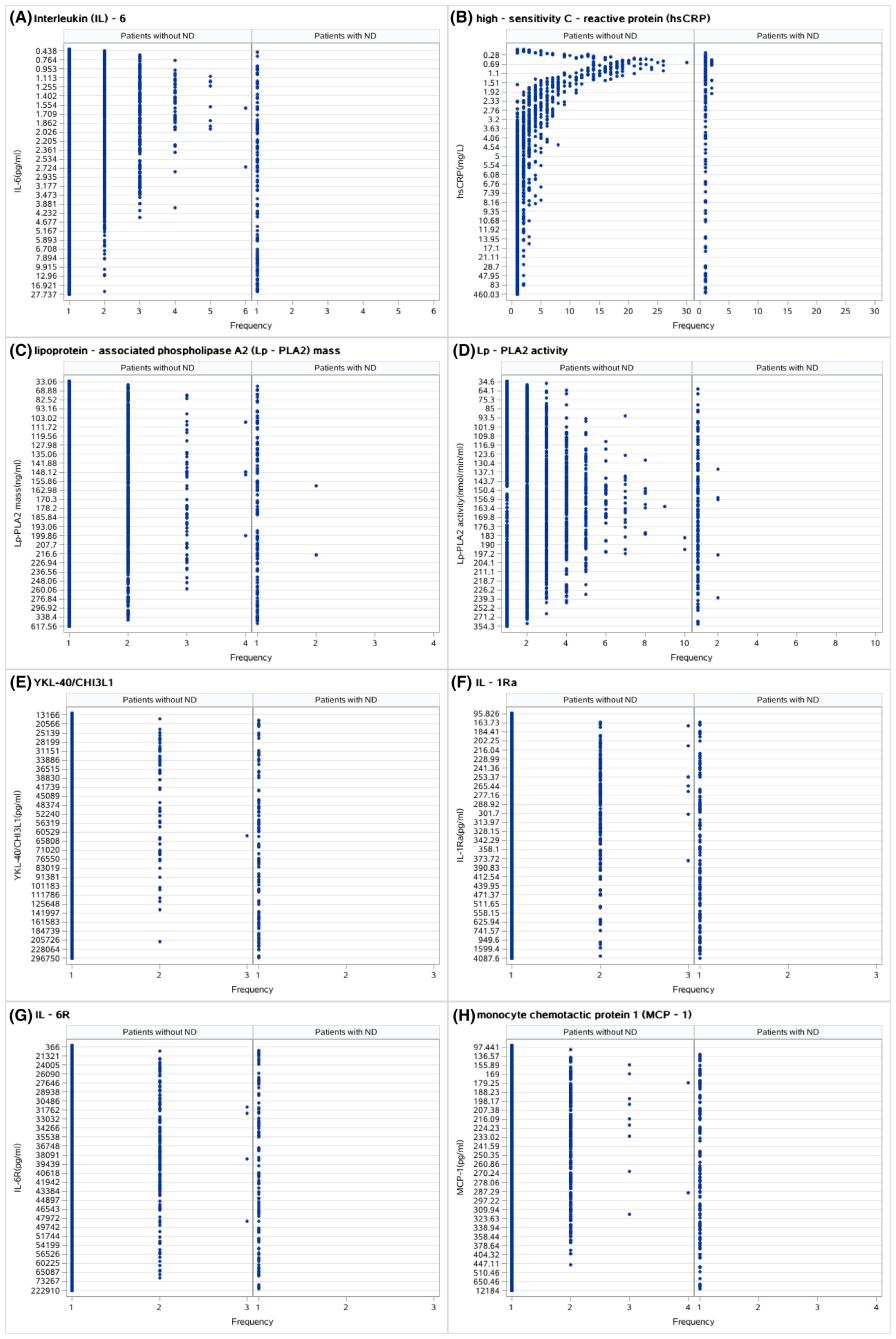
Dot plots based on neurological deterioration (ND) status. (A) Interleukin (IL)‐6, (B) high‐sensitivity C‐reactive protein (hsCRP), (C) lipoprotein‐associated phospholipase A2 (Lp‐PLA2) mass, (D) Lp‐PLA2 activity, (E) YKL‐40/CHI3L1, (F) IL‐1Ra, (G) IL‐6R, and (H) monocyte chemotactic protein 1 (MCP‐1).

In‐hospital ND occurred in 121 (3%) patients, and none of the enrolled patients experienced in‐hospital death. Patients with ND had higher levels of hsCRP (median, 1.5 vs. 2.0 mg/L), IL‐6 (median, 2.4 vs. 2.8 pg/mL), IL‐6R (median, 39784.0 vs. 40762.7 pg/mL), IL‐1Ra (median, 330.3 vs. 391.9 pg/mL), Lp‐PLA2 mass (median, 172.7 vs. 178.7 ng/mL), Lp‐PLA2‐A (median, 153.9 vs. 164.9 nmol/min/mL), and YKL‐40/CHI3L1 (median, 61819.4 vs. 76017.6 pg/mL), except for MCP‐1 (median, 308.1 vs. 296.4 pg/mL), than patients without ND (Table 2 and Figure 1).

**TABLE 2:** Inflammatory markers at admission by in‐hospital ND status.

Variables	No ND (*N* = 3910 [97.0%])	ND (*N* = 121 [3.0%])	ASD (%)
hsCRP (mg/L)			
Mean ± SD	5.5 ± 19.5	11.6 ± 30.5	23.8
Median (IQR)	1.5 (0.8–3.8)	2.0 (0.8–6.6)	0.4
IL‐6 (pg/mL)			
Mean ± SD	3.8 ± 4.0	5.7 ± 5.7	38.6
Median (IQR)	2.4 (1.5–4.2)	2.8 (1.9–8.1)	0.6
IL‐6R (pg/mL)			
Mean ± SD	41798.4 ± 15507.4	42479.5 ± 15081.5	4.5
Median (IQR)	39784.0 (31282.7–49741.5)	40762.7 (32201.2–52305.0)	626.7
IL‐1Ra (pg/mL)			
Mean ± SD	472.8 ± 515.3	550.2 ± 541.1	14.6
Median (IQR)	330.3 (250.0–469.7)	391.9 (287.6–565.3)	48.0
Lp‐PLA2 mass (ng/mL)			
Mean ± SD	180.2 ± 75.4	189.9 ± 75.9	12.8
Median (IQR)	172.7 (126.7–224.0)	178.7 (135.0–234.4)	9.1
Lp‐PLA2‐A (nmol/min/mL)			
Mean ± SD	159.9 ± 49.5	168.2 ± 46.6	17.3
Median (IQR)	159.3 (126.1–192.2)	164.9 (134.8–197.8)	7.7
YKL‐40/CHI3L1 (pg/mL)			
Mean ± SD	83869.9 ± 61471.7	101155.6 ± 68973.9	26.5
Median (IQR)	61819.4 (37589.1–112173.4)	76017.6 (48846.2–151053.0)	12,472
MCP‐1 (pg/mL)			
Mean ± SD	308.1 ± 302.9	296.4 ± 188.1	4.6
Median (IQR)	262.1 (202.6–337.3)	264.1 (198.5–329.9)	−5.2

Abbreviations: ASD, absolute standard difference; hsCRP, high‐sensitivity C‐reactive protein; IL‐1Ra, IL‐1 receptor antagonist; IL‐6, interleukin‐6; IL‐6R, IL‐6 receptor; IQR, interquartile range; Lp‐PLA2, lipoprotein‐associated phospholipase A2; Lp‐PLA2‐A, Lp‐PLA2 activity; MCP‐1, monocyte chemotactic protein 1; ND, neurological deterioration; SD, standard deviation; YKL‐40/CHI3L1, chitinase‐3‐like protein 1.

The associations of these inflammatory biomarkers with in‐hospital ND are further presented by quartiles and standard deviations (Table 3). After adjustment for age, sex, tobacco use, NIHSS scores at admission and discharge, SBP, lipid metabolism disorders, heart failure, peripheral arterial disease, carotid stenosis, infarction topography, offending circulation, and stroke etiology, both hsCRP levels (adjusted OR, 1.17 [95% CI, 1.06–1.31]) and IL‐6 levels (adjusted OR, 1.43 [95% CI, 1.24–1.66]) remained independent predictors of in‐hospital ND (Table 3). The above relationship was also observed in long‐term poor outcomes, including unfavorable functional outcomes (defined as mRS 2–5) and death (Table S2).

**TABLE 3:** Association between inflammatory markers at admission and in‐hospital ND.

Outcomes	No. of patients in strata	Event (%)	Crude OR (95% CI)	*p*‐value	Crude *p*‐value for trend	Adjusted OR (95% CI)	*p*‐value	Adjusted *p*‐value for trend
hsCRP (mg/L)					0.0571			0.2107
Q1	1001	31 (3.10)	Ref			Ref		
Q2	1009	20 (1.98)	0.63 (0.36–1.12)	0.1150		0.51 (0.26–0.99)	0.0478	
Q3	1012	25 (2.47)	0.79 (0.47–1.3)	0.3937		0.60 (0.32–1.15)	0.1240	
Q4	1009	45 (4.46)	1.46 (0.92–2.33)	0.1111		1.28 (0.75–2.18)	0.3676	
Per SD			1.14 (1.05–1.25)	0.0033		1.17 (1.06–1.31)	0.0031	
IL‐6 (pg/mL)					0.0017			0.0087
Q1	1007	20 (1.99)	Ref			Ref		
Q2	1007	28 (2.78)	1.41 (0.79–2.52)	0.2447		1.19 (0.58–2.45)	0.6338	
Q3	1009	27 (2.68)	1.36 (0.76–2.44)	0.3065		1.33 (0.66–2.68)	0.4267	
Q4	1008	46 (4.56)	2.36 (1.39–4.02)	0.0016		2.26 (1.16–4.41)	0.0172	
Per SD			1.37 (1.21–1.56)	<0.0001		1.43 (1.24–1.66)	<0.0001	
IL‐6R (pg/mL)					0.3409			0.7401
Q1	1008	27 (2.68)	Ref			Ref		
Q2	1008	31 (3.08)	1.15 (0.68–1.95)	0.5944		1.12 (0.62–2.03)	0.7172	
Q3	1008	27 (2.68)	1.00 (0.58–1.72)	1.0000		1.06 (0.58–1.95)	0.8518	
Q4	1007	36 (3.57)	1.34 (0.81–2.24)	0.2491		1.14 (0.62–2.08)	0.6793	
Per SD			1.04 (0.88–1.24)	0.6337		0.98 (0.80–1.21)	0.8717	
IL‐1Ra (pg/mL)					0.0061			0.2540
Q1	1007	20 (1.99)	Ref			Ref		
Q2	1008	26 (2.58)	1.31 (0.73–2.36)	0.3740		0.99 (0.51–1.94)	0.9840	
Q3	1008	36 (3.57)	1.83 (1.05–3.18)	0.0328		1.46 (0.79–2.71)	0.2272	
Q4	1008	39 (3.87)	1.99 (1.15–3.43)	0.0138		1.30 (0.69–2.44)	0.4225	
Per SD			1.13 (0.98–1.30)	0.1074		1.09 (0.91–1.30)	0.3624	
Lp‐PLA2 mass (ng/mL)					0.3042			0.4141
Q1	1007	25 (2.48)	Ref			Ref		
Q2	1008	32 (3.17)	1.29 (0.76–2.19)	0.3503		1.40 (0.75–2.62)	0.2903	
Q3	1008	30 (2.98)	1.21 (0.70–2.06)	0.4975		1.48 (0.78–2.79)	0.2222	
Q4	1008	34 (3.37)	1.37 (0.81–2.32)	0.2378		1.33 (0.71–2.51)	0.3767	
Per SD			1.13 (0.95–1.34)	0.1652		1.08 (0.89–1.33)	0.4359	
Lp‐PLA2‐A (nmol/min/mL)					0.0798			0.2604
Q1	1005	20 (1.99)	Ref			Ref		
Q2	1007	35 (3.48)	1.77 (1.02–3.09)	0.0436		1.96 (1.04–3.69)	0.0377	
Q3	1010	30 (2.97)	1.51 (0.85–2.67)	0.1600		1.54 (0.79–3.00)	0.2030	
Q4	1009	36 (3.57)	1.82 (1.05–3.17)	0.0337		1.70 (0.88–3.28)	0.1147	
Per SD			1.18 (0.99–1.41)	0.0683		1.15 (0.93–1.41)	0.1994	
YKL‐40/CHI3L1 (pg/mL)					0.0124			0.2887
Q1	1007	20 (1.99)	Ref			Ref		
Q2	1008	31 (3.08)	1.57 (0.89–2.77)	0.1225		1.52 (0.77–2.98)	0.2290	
Q3	1008	29 (2.88)	1.46 (0.82–2.60)	0.1967		1.31 (0.65–2.62)	0.4539	
Q4	1008	41 (4.07)	2.09 (1.22–3.60)	0.0076		1.60 (0.80–3.22)	0.1850	
Per SD			1.28 (1.09–1.50)	0.0027		1.18 (0.96–1.45)	0.1206	
MCP‐1 (pg/mL)					0.7075			0.8098
Q1	1007	32 (3.18)	Ref			Ref		
Q2	1008	28 (2.78)	0.87 (0.52–1.46)	0.5977		0.82 (0.45–1.49)	0.5090	
Q3	1008	34 (3.37)	1.06 (0.65–1.74)	0.8055		1.17 (0.67–2.04)	0.5801	
Q4	1008	27 (2.68)	0.84 (0.50–1.41)	0.5069		0.82 (0.45–1.49)	0.5087	
Per SD			0.94 (0.73–1.23)	0.6641		0.93 (0.69–1.27)	0.6587	

Abbreviations: CI, confidence interval; hsCRP, high‐sensitivity C‐reactive protein; IL‐1Ra, IL‐1 receptor antagonist; IL‐6, interleukin‐6; IL‐6R, IL‐6 receptor; Lp‐PLA2, lipoprotein‐associated phospholipase A2; Lp‐PLA2‐A, Lp‐PLA2 activity; MCP‐1, monocyte chemotactic protein 1; ND, neurological deterioration; OR, odds ratio; Q1–4, quartiles 1–4; SD, standard deviation; YKL‐40/CHI3L1, chitinase‐3‐like protein 1.

### 3.3 Incremental predictive value of inflammatory profiles

We evaluated whether hsCRP, IL‐6, both hsCRP and IL‐6, or all inflammatory markers combined would have an increased predictive value in addition to including conventional risk factors (Table 4). The IDI and category‐free NRI were 0.012 (*p* = 0.0017) and 0.329 (*p* = 0.0018), respectively, with the addition of IL‐6, which contributed the most to improving the risk classification algorithms. However, simultaneous addition of IL‐6 and hsCRP did not improve the prediction of in‐hospital ND over IL‐6 alone.

**TABLE 4:** Reclassification and discrimination statistics for in‐hospital ND status.

Predictors	AUC	*p*‐value for AUC difference	IDI	*p*‐value	NRI	*p*‐value
Conventional risk factors	0.691	–	–	–	–	–
hsCRP	0.702	0.1672	0.003	0.0668	0.180	0.0869
IL‐6	0.720	0.0623	0.012	0.0017	0.329	0.0018
IL‐1Ra	0.692	0.7201	0.000	0.4365	0.064	0.5407
Lp‐PLA2‐A	0.695	0.4931	0.001	0.1798	0.018	0.8655
YKL40/CHI3L1	0.696	0.5190	0.001	0.1801	0.095	0.3677
hsCRP+IL‐6	0.720	0.1815	0.009	0.0099	0.249	0.0181
All	0.723	0.0594	0.016	0.0007	0.308	0.0034

Abbreviations: hsCRP, high‐sensitivity C‐reactive protein; IDI, integrated discrimination improvement; IL‐1Ra, IL‐1 receptor antagonist; IL‐6, interleukin‐6; IL‐6R, IL‐6 receptor; Lp‐PLA2, lipoprotein‐associated phospholipase A2; Lp‐PLA2‐A, Lp‐PLA2 activity; MCP‐1, monocyte chemotactic protein 1; ND, neurological deterioration; NRI, net reclassification index; ROC, area under the curve; YKL‐40/CHI3L1, chitinase‐3‐like protein 1.

### 3.4 Subgroup analysis

Subgroup analysis by comparing patients with a stroke etiology of LAA and SVO showed consistent results with the main analysis (Table 5). We evaluated the incremental predictive value of IL‐6 and hsCRP in patients with LAA or SVO etiology as well (Table 6). The addition of IL‐6 contributed the most to improving the risk classification algorithms and was consistent with the results of the main analysis. As for patients with SVO etiology, adding IL‐6, hsCRP, or the two markers simultaneously did not improve the predictive effect compared with the conventional model.

**TABLE 5:** Inflammatory markers at admission and in‐hospital ND by stroke etiology subgroup.

Outcomes	No of patients in strata	Event (%)	Crude OR (95% CI)	*p*	Crude P for trend	Adjusted OR (95% CI)	*p*	Adjusted P for trend
LAA								
IL‐6					0.0411			0.0614
Q1	156	5 (3.21)	Ref			Ref		
Q2	197	6 (3.05)	0.949 (0.284–3.168)	0.9318		0.892 (0.191–4.165)	0.8845	
Q3	242	8 (3.31)	1.032 (0.332–3.215)	0.9560		1.106 (0.272–4.494)	0.8879	
Q4	250	18 (7.20)	2.343 (0.852–6.445)	0.0990		2.429 (0.651–9.056)	0.1863	
Per SD			1.579 (1.280–1.948)	<0.0001		1.620 (1.270–2.066)	0.0001	
hsCRP					0.0675			0.0693
Q1	158	4 (2.53)	Ref			Ref		
Q2	215	9 (4.19)	1.682 (0.509–5.563)	0.3942		3.185 (0.671–15.119)	0.1448	
Q3	216	7 (3.24)	1.289 (0.371–4.483)	0.6892		1.498 (0.268–8.381)	0.6453	
Q4	256	17 (6.64)	2.738 (0.904–8.292)	0.0747		4.516 (1.001–20.374)	0.0498	
Per SD			1.293 (1.096–1.526)	0.0023		1.267 (1.065–1.508)	0.0075	
SVO								
IL‐6					0.5050			0.5574
Q1	266	4 (1.50)	Ref			Ref		
Q2	272	6 (2.21)	1.477 (0.4125.296‐)	0.5490		1.210 (0.329–4.442)	0.7743	
Q3	245	5 (2.04)	1.365 (0.362–5.141)	0.6460		1.272 (0.327–4.940)	0.7286	
Q4	202	5 (2.48)	1.662 (0.441–6.271)	0.4531		1.517 (0.385–5.968)	0.5512	
Per SD			1.459 (1.078–1.975)	0.0145		1.427 (1.035–1.968)	0.0301	
hsCRP					0.8241			0.8948
Q1	299	9 (3.01)	Ref			Ref		
Q2	268	0 (0.00)	<0.001 (<0.001– > 999.999)	0.9430		<0.001 (<0.001– > 999.999)	0.9543	
Q3	238	8 (3.36)	1.121 (0.426–2.951)	0.8174		1.056 (0.385–2.898)	0.9158	
Q4	180	3 (1.67)	0.546 (0.146–2.044)	0.3691		0.598 (0.154–2.321)	0.4575	
Per SD			0.994 (0.645–1.531)	0.9774		0.999 (0.628–1.587)	0.9950	

Abbreviations: CI, confidence interval; hsCRP, high‐sensitivity C‐reactive protein; IL‐6, interleukin‐6; LAA, large‐artery atherosclerosis; ND, neurological deterioration; OR, odds ratio; Q1–4, quartiles 1–4; SVO, small‐vessel occlusion.

**TABLE 6:** Reclassification and discrimination statistics for in‐hospital ND status by stroke etiology subgroup.

	AUC	*p*‐value for AUC Difference	IDI	*p*‐value	NRI	*p*‐value
LAA						
conventional	0.63807	–	–	–	–	–
hsCRP	0.67324	0.1878	0.019922	0.1465	0.11148	0.5513
IL‐6	0.69454	0.1793	0.038193	0.0059	0.44838	0.0166
hsCRP+IL‐6	0.69911	0.1402	0.045592	0.0090	0.43501	0.0201
SVO						
conventional	0.79416	–	–	–	–	–
hsCRP	0.79405	0.3107	0.000001	0.9079	0.11965	0.5966
IL‐6	0.80060	0.6862	0.017284	0.1822	−0.019651	0.9307
hsCRP+IL‐6	0.80251	0.6222	0.021581	0.1922	0.047598	0.8332

Abbreviations: AUC, area under the curve; hsCRP, high‐sensitivity C‐reactive protein; IDI, integrated discrimination improvement; IL‐6, interleukin‐6; LAA, large‐artery atherosclerosis; ND, neurological deterioration; NRI, net reclassification index; SVO, small‐vessel occlusion.

## 4 DISCUSSION

In this hospital‐based, centralization‐tested prospective study, higher IL‐6 and hsCRP levels were associated with in‐hospital ND and adverse long‐term outcomes, including unfavorable functional outcomes and death at 3 months in patients with minor AIS. Elevated IL‐6 was an independent predictor for ND in minor AIS patients with LAA and SVO subtypes, whereas a rising hsCRP remained indicative only in the LAA subtype but not in the SVO subtype. IL‐6 had the most notable incremental predictive value of in‐hospital ND in addition to conventional predictors, both collectively and in the LAA subgroup.

Most hospitalized patients with ND have early ND (END), compared to delayed or late ND^3^; however, the time definition of END is still operational. Existing research suggests that patients who experience END are more likely to have poor long‐term outcomes.^3^, ^19^, ^24^ In our study, we defined ND as a 4‐point increase in the NIHSS score. This corresponds with the definition in most previous studies. However, our results showed a lower incidence of ND than in previous studies.^6^, ^25^, ^26^, ^27^, ^28^ This may be partly because of our selection of patients with minor strokes, and partly because we analyzed patients with ND at discharge; therefore, those who experienced a good rehabilitation process at a later stage were not included. We assumed that the identified inflammatory factors are more likely to be associated with non‐recovery, despite the patients being treated with the best medications. Moreover, higher levels of IL‐6 and hsCRP were related to long‐term adverse outcomes in patients with minor AIS. Independent associations of IL‐6 or YKL‐40 and poor functional outcomes in AIS patients have been found in a previous study.^29^ Our refined population‐based study provides evidence for stratified treatment for a more specific subgroup of patients to be monitored during hospitalization.

To provide an overall, holistic view of post‐stroke inflammation, multiple biomarkers were analyzed in this study. Independent associations were found between levels of IL‐6 or hsCRP tested within 24 h of onset, in‐hospital ND, and unfavorable function outcome and death at 3 months. High hsCRP concentrations are associated with the progression of arteriosclerosis in community‐based cohorts.^30^, ^31^ Thus, hsCRP could be considered a marker of systemic and vascular inflammation. IL‐6 shows a clear dose–response association with the risk of incident AIS.^32^ A previous study showed that over 80% of the functional damage after AIS resulted from perturbations to the IL‐6 pathway, rather than stroke recurrence.^33^ As ND is considered to be a predictive factor for long‐term poor outcomes,^7^ the correlation between ND and poor long‐term outcomes may result from a prolonged post‐stroke inflammatory state. IL‐6 signaling mechanisms^34^ and levels of hsCRP^35^ vary at different phases of an ischemic stroke; therefore, further studies are required to provide clear evidence of inflammatory profiles during admission, hospitalization, and follow‐up to verify this hypothesis.

In our study, patients with in‐hospital ND were found to have a stroke etiology of LAA and SVO. We compared the relationship between the two etiologies and IL‐6 or hsCRP. The independent association between higher hsCRP levels and the risk of ND was only observed in LAA stroke patients, but not in SVO stroke patients. Distributing atherosclerotic stenosis, occlusions, and LAA etiology were associated with END,^36^ and LAA proved to be a stronger activator of inflammation than SAO, with distinctive changes in CRP.^37^ Elevated IL‐6 levels remained an independent predictor for both etiologies, which was consistent with a previous study.^10^ The distinct inflammatory activation mechanisms of the various etiologies may explain the above differences.

This study elevated the incremental predictive values of inflammatory markers at admission for in‐hospital ND. It may advocate for more aggressive treatment for patients with mild neurological deficit symptoms. The ongoing CHANCE‐3 trial (colchicine in high‐risk patients with acute minor‐to‐moderate ischemic stroke or TIA) may provide stronger evidence of the effects of anti‐inflammatory therapies on inflammatory factors and ND prognosis.^38^

This study had several limitations. First, only biomarker measurements at admission were considered, precluding assessments of the changes in these markers. Second, the NIHSS score was measured only at admission and discharge; therefore, those with ND who subsequently recovered partially or completely during hospitalization were not included. However, this did not affect our results, as we aimed to identify patients who still had ND after treatment. Third, the mechanisms of ND can be categorized into ischemic progression, symptomatic hemorrhage, and brain edema^39^ and may differ in their time of occurrence. We did not distinguish between these types in our study. Thus, future studies should conduct subgroup analyses on the time of onset and stroke etiology and correlate them with multiple continuous biomarker measurements.

## 5 CONCLUSION

In conclusion, elevated hsCRP and IL‐6 levels indicated higher in‐hospital ND risk. Adding inflammatory cytokines significantly improved risk classification, with IL‐6 as the main contributor. Our findings comprehensively described the characteristics of post‐stroke systemic and vascular inflammation. They may offer evidence supporting the potential use of inflammatory parameters as biomarkers for predicting ND, guiding the implementation of personalized treatments in AIS.

## CONFLICT OF INTEREST STATEMENT

Yongjun Wang is an Editorial Board member of CNS Neuroscience and Therapeutics and a co‐author of this article. To minimize bias, he was excluded from all editorial decision‐making related to the acceptance of this article for publication in the manuscript under the disclosure section.

## Supporting information


Data S1.

## Data Availability

The data are available from the corresponding author upon reasonable request.
