# Supplementary material for: Inflammatory marker profiles and in‐hospital neurological deterioration in patients with acute minor ischemic stroke
Source: CNS Neurosci Ther. 2024 Mar 3;30(3):e14648. doi: 10.1111/cns.14648 (PMC10909616; doi:10.1111/cns.14648)
Supplement: Supplementary file 1 — Data S1. [file CNS-30-e14648-s001.docx]

| **Figure S1. Patient flowchart. CNSR-III, Third China National Stroke Registry; NIHSS, National Institutes of Health Stroke Scale.**  CNSR-III (n=15166)  Onset to door >24 h (n=5886)  NIHSS > 5 (n=2739)  Minor acute ischemic stroke patients (n=6541)  1. Patients received thrombolysis or thrombectomy (n=618)  2. Missing data on NIHSS at admission or at discharge (n=8)  3. Missing inflammatory marker data (n=1884)  Final included patients (n=4031)  **Table S1. Demographic and clinical characteristics of patients by inflammatory marker status.** | | | | | | | |
| --- | --- | --- | --- | --- | --- | --- | --- |
|  |  |  |  |  |  |  |  |
| **Variables** | | **Total (N=5915 [100%])** | **No Inflammatory Marker (N=1884 [31.9%])** | **Inflammatory Marker (N=4031 [68.1%])** | **P Value** | **ASD** |  |
| **Demographics** | |  |  |  |  |  |  |
| **Age in years** | |  |  |  | 0.1432 |  |  |
| Mean±SD | | 62.1±11.3 | 61.7±11.2 | 62.2±11.3 |  | 4.4 |  |
| Min–Max | | 23.0–96.0 | 23.0–96.0 | 24.0–95.0 |  |  |  |
| Median (IQR) | | 62.0 (54.0–70.0) | 62.0 (53.0–69.0) | 62.0 (54.0–70.0) |  | 0.0 |  |
| **Female** | | 1824 (30.8) | 578 (30.7) | 1246 (30.9) | 0.8577 | 0.4 |  |
| **Smoking** | | 1872 (31.6) | 569 (30.2) | 1303 (32.3) | 0.1020 | 4.5 |  |
| **NIHSS at admission** | |  |  |  | 0.6292 |  |  |
| Mean±SD | | 2.2±1.6 | 2.1±1.6 | 2.2±1.6 |  | 6.3 |  |
| Min–Max | | 0.0–5.0 | 0.0–5.0 | 0.0–5.0 |  |  |  |
| Median (IQR) | | 2.0 (1.0–3.0) | 2.0 (1.0–3.0) | 2.0 (1.0–4.0) |  | 0.0 |  |
| **NIHSS** | |  |  |  | 0.0103 | 7.2 |  |
| NIHSS, <=3 | | 4453 (75.3) | 1458 (77.4) | 2995 (74.3) |  |  |  |
| NIHSS, 4-5 | | 1462 (24.7) | 426 (22.6) | 1036 (25.7) |  |  |  |
| **SBP** | |  |  |  | 0.5320 |  |  |
| Mean±SD | | 150.7±22.0 | 150.4±21.4 | 150.8±22.3 |  | 1.8 |  |
| Min–Max | | 85.0–247.5 | 92.5–247.5 | 85.0–241.5 |  |  |  |
| Median (IQR) | | 149.0 (135.0–164.0) | 148.0 (135.5–162.8) | 149.0 (135.0–164.5) |  | 0.0 |  |
| **DBP** | |  |  |  | 0.9305 |  |  |
| Mean±SD | | 87.6±13.0 | 87.5±12.5 | 87.6±13.2 |  | 0.8 |  |
| Min–Max | | 47.5–154.0 | 50.0–154.0 | 47.5–148.0 |  |  |  |
| Median (IQR) | | 86.0 (79.5–95.5) | 86.0 (80.0–95.5) | 86.5 (79.0–95.5) |  | 0.0 |  |
| **Medical history** | |  |  |  |  |  |  |
| **Prior stroke/TIA** | | 1483 (25.1) | 478 (25.4) | 1005 (24.9) | 0.7162 | 1.2 |  |
| **Hypertension** | | 3682 (62.2) | 1164 (61.8) | 2518 (62.5) | 0.6140 | 1.4 |  |
| **Diabetes mellitus** | | 1336 (22.6) | 405 (21.5) | 931 (23.1) | 0.1706 | 3.8 |  |
| **Lipid metabolism disorders** | | 489 (8.3) | 131 (7.0) | 358 (8.9) | 0.0121 | 7.0 |  |
| **Prior CHD/MI** | | 656 (11.1) | 221 (11.7) | 435 (10.8) | 0.2840 | 2.8 |  |
| **Atrial fibrillation/flutter** | | 349 (5.9) | 88 (4.7) | 261 (6.5) | 0.0061 | 7.8 |  |
| **Heart failure** | | 29 (0.5) | 6 (0.3) | 23 (0.6) | 0.1959 | 4.5 |  |
| **Peripheral arterial disease** | | 55 (0.9) | 13 (0.7) | 42 (1.0) | 0.1889 | 3.3 |  |
| **Carotid stenosis** | | 47 (0.8) | 11 (0.6) | 36 (0.9) | 0.2121 | 3.5 |  |
| **Infarction pattern** | |  |  |  | 0.4197 | 4.1 |  |
| None | | 1628 (27.5) | 524 (27.8) | 1104 (27.4) |  | 0.9 |  |
| Single infarction | | 2319 (39.2) | 714 (37.9) | 1605 (39.8) |  | 3.9 |  |
| Multiple infarction | | 1904 (32.2) | 628 (33.3) | 1276 (31.7) |  | 3.4 |  |
| Watershed infarction | | 64 (1.1) | 18 (1.0) | 46 (1.1) |  | 1.0 |  |
| **Infarction circulation** | |  |  |  | 0.9392 | 1.3 |  |
| None | | 1628 (27.5) | 524 (27.8) | 1104 (27.4) |  | 0.9 |  |
| Anterior circulating infarction | | 2572 (43.5) | 821 (43.6) | 1751 (43.4) |  | 0.4 |  |
| Posterior circulation infarction | | 1448 (24.5) | 458 (24.3) | 990 (24.6) |  | 0.7 |  |
| Anterior and posterior circulatory infarction | | 267 (4.5) | 81 (4.3) | 186 (4.6) |  | 1.5 |  |
| **Stroke etiology** | |  |  |  | 0.1201 | 6.9 |  |
| LAA | | 1257 (21.3) | 412 (21.9) | 845 (21.0) |  | 2.2 |  |
| CE | | 340 (5.7) | 89 (4.7) | 251 (6.2) |  | 6.6 |  |
| SAO | | 1439 (24.3) | 454 (24.1) | 985 (24.4) |  | 0.7 |  |
| Other | | 2879 (48.7) | 929 (49.3) | 1950 (48.4) |  | 1.8 |  |
|  | | | | | | |  |
|  | | | | | | | |

ND, neurological deterioration; NIHSS, National Institutes of Health Stroke Scale; SBP, systolic blood pressure; DBP, diastolic blood pressure; TIA, transient ischaemic attack; CHD, coronary heart disease; MI, myocardial infarction; LAA, large-artery arteriosclerosis; CE, cardioembolic stroke; SAO, small-artery occlusion; SD, standard deviation; IQR, interquartile range; ASD, absolute standard difference.

**Table S2. Inflammatory markers and poor functional outcomes at 3 months**

| Biomarkers | Outcomes | per | No of patients in strata | Event (%) | Crude OR  (95% CI) | P | Crude P for trend | Adjusted OR  (95% CI) | P | Adjusted P for trend |
| --- | --- | --- | --- | --- | --- | --- | --- | --- | --- | --- |
| IL-6  (pg/mL) | mRS score 2-5 |  |  |  |  |  | <.0001 |  |  | <.0001 |
|  |  | Q1 | 998 | 32(3.21) | Ref |  |  | Ref |  |  |
|  |  | Q2 | 1003 | 41(4.09) | 1.286  (0.803-2.060) | 0.2946 |  | 1.067  (0.619-1.841) | 0.8144 |  |
|  |  | Q3 | 996 | 76(7.63) | 2.493  (1.634-3.805) | <.0001 |  | 1.618  (0.980-2.671) | 0.0599 |  |
|  |  | Q4 | 991 | 121(12.21) | 4.198  (2.813-6.2) | <.0001 |  | 2.574  (1.589-4.169) | 0.0001 |  |
|  |  | Per SD |  |  | 1.483  (1.357-1.621) | <.0001 |  | 1.381  (1.236-1.542) | <.0001 |  |
|  | death |  |  |  |  |  | 0.0001 |  |  | 0.0181 |
|  |  | Q1 | 1007 | 3(0.30) | Ref |  |  | Ref |  |  |
|  |  | Q2 | 1007 | 2(0.20) | 0.666  (0.111-3.985) | 0.6559 |  | 0.515  (0.085-3.104) | 0.4686 |  |
|  |  | Q3 | 1009 | 2(0.20) | 0.667  (0.112-3.994) | 0.6578 |  | 0.000  (0.000) | 0.9892 |  |
|  |  | Q4 | 1008 | 19(1.88) | 6.392  (1.892-21.600) | 0.0028 |  | 2.745  (0.752-10.013) | 0.1263 |  |
|  |  | Per SD |  |  | 2.008  (1.655-2.435) | <.0001 |  | 2. 005  (1.590-2.529) | <.0001 |  |
| Hs-CRP  (mg/L) | mRS score 2-5 |  |  |  |  |  | <.0001 |  |  | 0.0393 |
|  |  | Q1 | 998 | 52(5.21) | Ref |  |  | Ref |  |  |
|  |  | Q2 | 998 | 53(5.31) | 1.020  (0.689-1.512) | 0.9201 |  | 1.038  (0.664-1.623) | 0.8693 |  |
|  |  | Q3 | 1000 | 57(5.70) | 1.100  (0.747-1.619) | 0.6301 |  | 0.927  (0.589-1.459) | 0.7445 |  |
|  |  | Q4 | 992 | 108(10.89) | 2.223  (1.577-3.133) | <.0001 |  | 1.520  (1.015-2.274) | 0.0419 |  |
|  |  | Per SD |  |  | 1.153  (1.070-1.242) | 0.0002 |  | 1.084  (0.982-1.197) | 0.1107 |  |
|  | death |  |  |  |  |  | 0.0010 |  |  | 0.0125 |
|  |  | Q1 | 1001 | 2(0.20) | Ref |  |  | Ref |  |  |
|  |  | Q2 | 1009 | 4(0.40) | 1.997  (0.366-10.902) | 0.4246 |  | 4.137  (0.460-37.206) | 0.2052 |  |
|  |  | Q3 | 10121 | 5(0.49) | 2.490  (0.483-12.835) | 0.2755 |  | 2.746  (0.284-26.601) | 0.3832 |  |
|  |  |  |  |  |  |  | <.0001 |  |  | 0.0393 |
|  |  | Q1 | 998 | 52(5.21) | Ref |  |  | Ref |  |  |

ND, neurological deterioration; hsCRP, high-sensitivity C-reactive protein; OR, odds ratio; CI, confidence interval; Q1–4, quartiles 1–4
